# Facebook Reviews as a Supplemental Tool for Hospital Patient Satisfaction and Its Relationship with Hospital Accreditation in Malaysia

**DOI:** 10.3390/ijerph18147454

**Published:** 2021-07-13

**Authors:** Afiq Izzudin A. Rahim, Mohd Ismail Ibrahim, Kamarul Imran Musa, Sook-Ling Chua

**Affiliations:** 1 Department of Community Medicine, School of Medical Science, Universiti Sains Malaysia, Kubang Kerian, Kota Bharu 16150, Kelantan, Malaysia; drafiqrahim@student.usm.my (A.I.A.R.); drkamarul@usm.my (K.I.M.); 2 Faculty of Computing and Informatics, Persiaran Multimedia, Multimedia University, Cyberjaya 63100, Selangor, Malaysia; slchua@mmu.edu.my

**Keywords:** social media, Facebook, patient satisfaction, quality of care, online review, accreditation, hospital management, clinical quality, Spearman correlation, Malaysia

## Abstract

Patient satisfaction is one indicator used to assess the impact of accreditation on patient care. However, traditional patient satisfaction surveys have a few disadvantages, and some researchers have suggested that social media be used in their place. Social media usage is gaining popularity in healthcare organizations, but there is still a paucity of data to support it. The purpose of this study was to determine the association between online reviews and hospital patient satisfaction and the relationship between online reviews and hospital accreditation. We used a cross-sectional design with data acquired from the official Facebook pages of 48 Malaysian public hospitals, 25 of which are accredited. We collected all patient comments from Facebook reviews of those hospitals between 2018 and 2019. Spearman’s correlation and logistic regression were used to evaluate the data. There was a significant and moderate correlation between hospital patient satisfaction and online reviews. Patient satisfaction was closely connected to urban location, tertiary hospital, and previous Facebook ratings. However, hospital accreditation was not found to be significantly associated with online reports of patient satisfaction. This groundbreaking study demonstrates how Facebook reviews can assist hospital administrators in monitoring their institutions’ quality of care in real time.

## 1. Introduction

Accreditation has gained global recognition as a framework for healthcare organizations to maintain quality of care. In an ideal environment, accreditation guarantees conformity with standards while promoting continuous quality improvement. Numerous kinds of healthcare accreditation exist for condition- or specialty-specific hospital and organization-level operations. The current state of knowledge about accreditation paints a mixed picture of whether it actually improves clinical processes and outcomes. A patient satisfaction score is a critical indicator of the quality of treatment and impact of accreditation in a healthcare setting [1]. Structured patient satisfaction surveys, such as the Hospital Consumer Assessment of Healthcare Providers and Systems (HCAHPS) and SERVQUAL (which measures service quality), are frequently used for assessing customer satisfaction with specific service quality criteria [2,3,4]. Even though these surveys are conducted in a systematic manner and capable of capturing a significant number of patient responses in a given hospital, they are expensive to implement, time intensive, and suffer from poor response rates and other challenges [2,5]. Thus, the internet, and social media specifically, has been proposed as a method of complementing or replacing traditional methods of assessing patient satisfaction and monitoring the quality of healthcare services [6,7].

Social media platforms such as Facebook and Twitter enable patients and the general public to share healthcare experiences and participate in real-time public conversation with healthcare professionals. Interactions between healthcare practitioners and patients can result in significant changes, combining patient-centered care, the internet, and social media—creating a ‘perfect storm’ environment [8]. Public or private healthcare issues will be discussed on social media channels based on customer feedback. The use of data in social media research is rapidly increasing in many areas of medicine and the health sciences. The widespread use of social media and the strength of word-of-mouth advertising may assist healthcare providers in monitoring their quality of care and identifying factors associated with patient satisfaction online, while also assisting patients in deciding where to obtain services and what to expect from a given hospital [9,10].

Patient online reviews through social media have developed into a patient-driven alternative that may offer near-instant feedback on a health care provider’s performance. The increasing knowledge base on the impact of online reviews on patients’ health care decision-making has resulted in an increase in the number of research papers on online reviews and social media [11]. Several studies indicated that online review and social media research possess greater scientific value to explore. Few research works have examined the relationship between online review sites and hospital quality indicators [12,13] or conventional patient satisfaction surveys [6,14]. Meanwhile, other researchers examined the quality of online reviews in relation to public perceptions and sentiments [2,15,16,17]. However, more empirical research beyond descriptive analyses are necessary to elucidate clinical and policy significance [11].

The present field of study about the use of social media in healthcare and its impact on healthcare remains in its infancy. When compared to the exponential rise of online review usage, the number of published research was modest particularly in developing countries [18,19]. Additionally, there is limited research examining the use of social media as a complement to hospital patient satisfaction surveys, and no study has yet examined the impact of accreditation on social media [11]. Thus, we seek to determine the relationship between hospital patient satisfaction surveys and online patient satisfaction as measured by Facebook reviews on the official Facebook pages of Malaysian public hospitals. Additionally, we are interested in investigating the link, if any, between hospital accreditation and online patient satisfaction as measured by Facebook reviews.

## 2. Theoretical Background

### 2.1. Hospital Facebook Reviews

The global population’s affinity for social media has recently prompted many healthcare organizations to use their country’s most popular social media platforms as a means of online communication and interaction with the public. A nationwide study in Taiwan revealed that Facebook enjoys high penetration and popularity in that country, which may have been one reason for more than half of Taiwan’s hospitals to establish an official Facebook page [20]. Facebook is also a vital component of social media use in Malaysia. According to a 2020 report, Facebook was used by 91.7% of Malaysian internet users and is expected to remain the country’s most popular social networking site [21].

While Facebook and other social media platforms have been shown to improve health outcomes through health education and information [18,19] and have proven beneficial during public health crises [22,23], other studies have examined specific features of social media platforms such as reviews and ratings and their relationship to patient satisfaction and hospital quality indicators [11]. For instance, Facebook includes a review tool that enables users to write narrative evaluations and rate the performance of businesses and institutions on those organizations’ Facebook pages. Numerous studies have discovered a low to moderate connection between Facebook evaluations and metrics from systematic patient satisfaction surveys [12,13,24], while another study found that clinical quality indicators such as reduced readmission rates are linked with patient recommendations and higher Facebook ratings [25]. According to a recent study, hospitals with an active Facebook page had more “likes”, a higher rate of patients willing to recommend the hospital, and a better overall satisfaction score [26]. Additional research on the patient perspective and its connection to hospital patients’ overall reviews on Facebook found links with many topics, including waiting times, treatment efficacy, and communication [16]. Thus, the popularity of Facebook among Malaysians and the Facebook review function provide an excellent opportunity for us to further explore its use for healthcare and the public good in Malaysia.

### 2.2. Hospital Accreditation Standards

Several hospital accreditation standards exist, including the Joint Commission International (JCI) standard developed in the United States, Accreditation Canada, and the Australian Council on Health Care Standards. Other standards include those established by the International Organization for Standardization, Six Sigma, Quality Awards, and the European Foundation for Quality Management. Meanwhile, Malaysia its own Malaysian Hospital Accreditation Program that is administered by the Malaysian Society for Health Quality (MSQH). A few countries or organizations have established certification systems that are adaptable to local requirements and circumstances based on mature accreditation models’ experiences [27]. For instance, in response to the global growth of Islamic medical tourism, researchers have proposed the creation of an international Islamic accreditation standard [28].

Assessing hospital accreditation standards is critical for ensuring the high quality, safety, and efficacy of healthcare services in hospitals. The efficacy of an accreditation system is contingent on the suitability, quality, and consistency of its procedures, standards, and surveyors. According to hospital administrators in Iran, decreasing the number of standards and criteria while increasing transparency may improve the accreditation process’s efficiency [29]. This finding was corroborated by a Brazilian study that identified leadership action as a key element in the certification process [30].

Apart from the standard evaluation, studies have revealed that hospital accreditation has a positive effect on organizational processes and structures, enhancing the safety and quality culture, improving patient care, and developing professionalism and staff competencies [1,31,32,33]. However, other research has shown that when an accreditation program was implemented in hospitals, there was no change in quality improvement, clinical treatment, or patient satisfaction [34,35]. What is most important to patients is that accreditation results in better patient care. Establishing a connection between accreditation and increased satisfaction or experience would increase patients’ confidence in and likelihood of choosing a recognized hospital [36].

### 2.3. Hospital Patient Satisfaction

For years, academics have evaluated hospital patient satisfaction using a variety of methods and conceptual frameworks. Earlier research indicated that patients with modest expectations were most satisfied, whereas those with unrealistic expectations were least satisfied [37]. When patients’ expectations matched the delivery of health services, they expressed satisfaction with those services [38]. Since those earlier efforts, the number of variables associated with patient satisfaction has grown and varies significantly in different studies [1,31,38]. However, one systematic study concluded that two powerful predictors of patient satisfaction are healthcare provider-related factors and patient-related characteristics [38]. That review found provider–related factors to be the greatest predictor of patient satisfaction across trials. Nine determinants of healthcare services were identified: technical care, interpersonal care, physical environment, accessibility, availability, financial resources, organizational features, continuity of treatment, and outcome of care. Among service-related variables, interpersonal skills and technical care characteristics had the greatest positive correlations.

Patient characteristics such as age, gender, education, socioeconomic status, marital status, race, religion, geographic characteristics, visit frequency, length of stay, health status, personality, and expectations were all investigated to determine their associations with patient satisfaction [35]. However, throughout the sample, these correlations were weak and inconsistent. As a result, the study suggested that it may be worth trying to construct patient satisfaction using quality indicators for health services and how people improve their satisfaction with health services. SERVQUAL and HCAHPS are two examples of structured surveys that are based on the quality of healthcare services. Patient satisfaction survey results can be very beneficial to both healthcare professionals and patients. They assist healthcare professionals in identifying areas of their services that may benefit from improvement. Increased patient satisfaction with healthcare services improves patient response to public hospitals [39]. According to studies, satisfied patients are more likely to adhere to their doctors’ suggested treatments and carry out follow-up visits, leading to improved health outcomes and recommendations of the hospital to others [38].

### 2.4. Hospital Accreditation and Patient Satisfaction Relationship

Although accreditation standards have been employed for decades and their effect on healthcare safety and quality has been widely acknowledged, attempts to assess the linkage between accreditation and patient satisfaction have produced varied results [31]. Earlier research established that accreditation was not linked with patient satisfaction [40,41] and that there was no statistically significant difference in patient satisfaction or recommendation between accredited and non-accredited hospitals [42]. This finding was supported by a study conducted in Lebanon in which the majority of patients expressed dissatisfaction with the quality of services [43], a study conducted in the United States in which no significant difference in patient satisfaction was found between accredited hospitals and other organizations [44], and an Iranian study in which an inverse relationship between patient satisfaction and quality of care was discovered [45]. However, many other studies have shown a positive correlation between accreditation and patient satisfaction in several settings, including Southeast Asia [46] and the Middle East [47,48].

### 2.5. Conceptual Background

**Figure 1:**
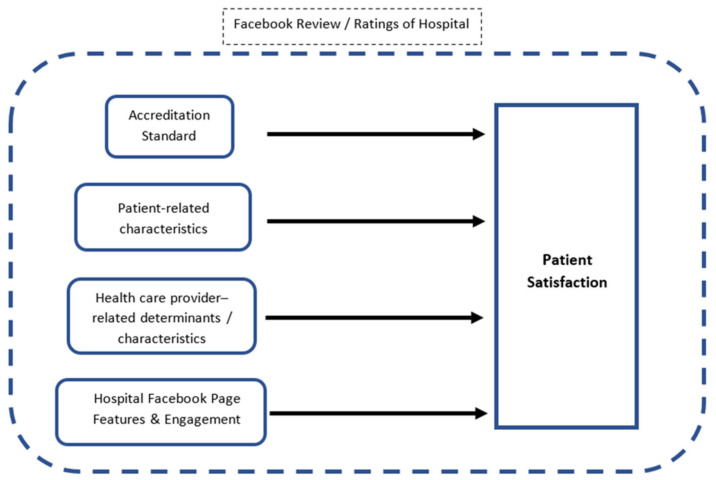
Conceptual framework of the study.

Our study generally synthesized key results or conceptual frameworks from literature studies on patient satisfaction-related variables. There are four major factors (accreditation status, patient related characteristics, healthcare provider related determinants, and Facebook page features or engagement) that may influence patient satisfaction in hospital’s Facebook reviews. Figure 1 illustrates the conceptual framework for this study.

## 3. Materials and Methods

This cross-sectional study of government hospitals in Malaysia was conducted from March 2020 to May 2021 to reconcile the topic’s homogeneity with the generalizability of the results. Universal sampling was employed.

### 3.1. Facebook Data

In the fall of 2020, we gathered data from the official Facebook pages of Malaysian public hospitals from 2018 to 2019. We began by using the Google search engine to browse hospital websites, using a list of all public hospitals in Malaysia obtained from the country’s Ministry of Health (MOH). We looked for URLs and links to each hospital’s official Facebook page. If the hospital’s website did not have a link to an official Facebook page, we continued our search on Facebook itself. When we discovered an official hospital Facebook page, we validated the information by using the hospital’s website’s address, contacting hospital administrators, or referring to our operating definition of an official hospital Facebook page. These search methods have also been applied in previous studies [12,13,23].

We defined an “official” hospital Facebook page as one with a “verified” symbol [49], one that used the hospital’s official name on the Facebook page, one with the hospital’s official name mentioned in the Facebook page’s description, or one with a Facebook page linked directly from the hospital’s official website. We included only publicly accessible Facebook pages that were linked with the hospital, and all data acquired from the official Facebook page were retained in a pro forma checklist, such as the average number of stars it earned and the inclusion of complete hospital information on the page. The hospital departments’ Facebook pages were eliminated, as were the pages of health institutions such as the MOH and the Institute of Medical Research and non-governmental organization hospitals and long-term care facilities.

### 3.2. Hospital Data

#### 3.2.1. Hospital Accreditation

The MSQH provided a list of accredited public hospitals in 2018 and 2019. MSQH is a not-for-profit organization founded in cooperation with the Malaysian MOH, the Malaysian Association of Private Hospitals, and the Malaysian Medical Association. Its mission is to enhance the quality of healthcare in Malaysia by improving organizational performance and patient care. MSQH is the only accreditor in Malaysia. Its certification standards address a broad variety of quality attributes, including treatment access, appropriateness, effectiveness, and safety, along with patient-centered activities, efficiency, and governance [50]. Safety is a key component of the standards; an entity that complies with all other criteria while failing to satisfy safety requirements will be refused certification. MSQH standards apply to all kinds of hospitals undergoing consideration for accreditation, whether public or private, large or small. A hospital seeking accreditation must perform a self-assessment prior to the accreditation survey. A team of surveyors conducts the assessment, and their report is then evaluated and voted on by members of the Malaysian Council for Health Care Standards. Malaysia had 69 certified public hospitals in both 2018 and 2019.

#### 3.2.2. Patient Satisfaction Survey

The MOH conducts a yearly survey of patient satisfaction in all public hospitals to establish a benchmark for quality hospital services. The survey is based on the SERVQUAL questionnaire; each hospital’s quality unit collects data and sends them to the MOH in Putrajaya for analysis. The survey is supplied to patients upon admission and collected prior to discharge. Satisfaction is evaluated by comparing the quality of the services to the patient’s expectations. SERVQUAL is linked to customer expectations before and during service delivery and to their perceptions of service quality after it has been delivered. A positive SERVQUAL difference indicates that a patient was pleased and that his or her expectations were fulfilled. Negative SERVQUAL results, on the other hand, indicate discontent, such as when a service is not finished completely. While those data are not publicly accessible, they are available for study at the MOH’s Medical Division in Putrajaya. However, due to technical issues, the MOH permitted us to examine only overall patient satisfaction data from 2018 and 2019 for each hospital, rather than the entire SERVQUAL domain. A hospital-wide patient satisfaction survey is one of the performance criteria used to assess service standards in the MSQH certification process. It serves as a proxy for determining the quality of patient-centered services and patient satisfaction [50]. There is no specific survey a hospital must conduct to ensure compliance with service standards. As a result, public hospitals often use the MOH patient satisfaction survey as part of the accreditation process [50].

### 3.3. Outcomes: Patient Satisfaction in Facebook Reviews

Users may employ the Facebook review feature to leave narrative reviews on the Facebook pages of organizations and companies. Since its debut in 2013, the Facebook review section has been included on the Facebook pages of many hospitals and is increasingly being used by patients and their families. Facebook had a five-star rating system until early 2018, when it switched to a binary approach—“Recommends” or “Does Not Recommend”—that significantly simplified the review process for Facebook. As with other social media platforms, Facebook reviews provide insights into how key stakeholders (e.g., former, and present patients, their relatives, or friends, past or current employees, and so on) perceive healthcare services. Numerous studies have already been conducted to evaluate Facebook reviews or ratings of hospital services and patient satisfaction or quality measurements [12,13,16]. To determine patient satisfaction, we used the Web Harvey (SysNucleus, Kochi, India) software package to collect customer recommendations in the reviews area of hospitals’ Facebook pages between January 2018 and December 2019. We define patient satisfaction as a recommendation in the review area of a given hospital’s Facebook page. However, suggestions made on non-Facebook review sites were excluded.

### 3.4. Statistical Analysis

Categorical data were given as frequencies and percentages for statistical analysis, while numerical data were provided as medians (interquartile range [IQR]) due to a non-normal distribution of the data. To determine the validity of customer recommendations in Facebook reviews as a supplementary tool for traditional patient satisfaction surveys, we compared the degree of hospital patient satisfaction as measured by the MOH survey to the proportion of patient recommendations on the hospital’s Facebook page. From the 2018 and 2019 datasets, we estimated the average percentage of patient satisfaction surveys and the proportion of Facebook recommendations for each institution. We then assessed their association using Spearman’s rank correlation coefficient. Correlations below 0.2 were considered weak, those between 0.2 and 0.5 were considered moderate, and those greater than 0.5 were considered high. Later, we used binary logistic regression analysis to determine the relationship with overall customer recommendations in Facebook reviews. The relationships were controlled for hospital factors (region, bed count, urban or rural location, and hospital type) and Facebook page features such as past star ratings, acceptable hospital information on the Facebook page, and administrator reaction in the Facebook review area. Previous research indicates that these characteristics are related to patient satisfaction. The findings were discussed in terms of those that were statistically significant at *p* ≤ 0.05. All statistical test assumptions were verified and fulfilled. To confirm the model fitness of our analysis, the Hosmer and Lemeshow test and the area under the operating ROC curve were used. SPSS, version 26 (IBM Corp., Armonk, NY, USA) software [51] was used to analyze the data.

## 4. Results

### 4.1. Hospital and Facebook Characteristics

In total, 86 of Malaysia’s 135 public hospitals (63.7%) had an official Facebook page, with 48 (55.5%) allowing consumer feedback on that platform. Accreditation had been granted to 25 (52.08%) of the 48 hospitals with Facebook reviews. Except for the western region, each region in Malaysia had at least 10 hospitals offering a Facebook review function: 37.5% of tertiary hospitals, 8.3% percent of secondary hospitals, and 54.2% percent of primary hospitals nationwide had Facebook review sections. The majority of these hospitals were located in urban areas and had an average of 730 beds. According to the annual MOH study, the average percentage (IQR in parentheses) of patients satisfied with treatment received in public hospitals was 96.93% (3.00). The average number of reviews per hospital Facebook page was 15.5 (27.5), and the average previous star rating was 5.00 (1.65). Many hospitals’ Facebook pages have contact information and responded to user reviews. The average proportion of customer recommendations in Facebook reviews was 80.7% (48.43). The hospitals and their Facebook characteristics are summarized in Table 1.

### 4.2. Correlation of Patient Satisfaction in Facebook Reviews and from Annual Hospital Surveys

The Spearman rank correlation indicated that the average proportion of patient satisfaction from the annual MOH survey was significantly correlated to the average proportion of patient recommendations in Facebook reviews (*r* = 0.35, *p* = 0.02, *n* = 48). We consider this correlation to be moderate.

### 4.3. Patient Satisfaction in Facebook Reviews and Its Associations

For the purpose of analyzing patient satisfaction, a total of 2019 Facebook reviews were collected from 48 hospital Facebook pages. The majority (49.1%) came from the western region, urban hospitals (87.1%), and tertiary facilities (88.5%); 9.1% of Facebook reviews received individualized feedback from hospital management. Approximately 61% of the reviews involved accredited hospitals. The majority of Malaysia’s public hospitals with the Facebook review feature enabled were recommended in Facebook reviews by patients or their families (74.4%). The Facebook reviews and their characteristics are summarized in Table 2.

### 4.4. Hospital Accreditation and Patient Satisfaction

Hospitals in northern (Odd ration (OR) 1.66, 95% Confident interval (CI): 1.12, 2.47), southern (OR 0.54, 95% CI: 0.34, 0.83), and eastern (OR 0.49, 95% CI: 0.32, 0.76) Malaysia exhibit significant relationships with patient satisfaction (*p* < 0.05). Hospitals located in urban areas (OR 1.85, 95% CI: 1.40, 2.43) and classified as tertiary (OR 1.62, 95% CI: 1.12, 2.35) were also significantly associated with patient satisfaction in Facebook reviews. Another significant link was with prior Facebook ratings (OR 1.14, 95% CI: 1.06, 1.23). There was, however, no significant association between hospital accreditation and patient satisfaction (OR 1.03, 95% CI: 0.84, 1.26). All relevant confounders and factors with *p*-values less than 0.25 were entered into the SPSS software during the multivariate analysis to build a final model for a confirmatory study of hospital accreditation. When geographical characteristics and previous Facebook ratings were controlled for, there was no significant association between hospital accreditation and patient satisfaction in Facebook reviews (AOR 0.95, 95% CI: 0.77, 1.17; *p* = 0.63). The fitness tests conducted on the models were judged to be satisfactory. Table 3 and Table 4 illustrate the analysis.

## 5. Discussion

This is the first study we are aware of that examines Facebook reviews as a tool for patient satisfaction and the impact of hospital accreditation on patient satisfaction expressed on social media platforms in Southeast Asia, and possibly across Asia.

### 5.1. Facebook Reviews and Patient Satisfaction Surveys

Social media use is growing among Malaysia’s public hospitals, the majority of which now have their own Facebook page. The results corroborated those of research in Taiwan demonstrating that the popularity of Facebook led to healthcare organizations’ desire to establish their own accounts on the site [20]. However, half of the Malaysian hospitals’ Facebook pages do not have a section for consumer feedback. It is unclear whether hospital administrators actively chose to disable feedback or were simply ignorant of the Facebook review function.

We discovered a moderate association between hospital patient satisfaction and consumer recommendations in Facebook reviews, which may offer information on service quality and patient experiences to hospital management. Previous research has shown a connection of low to moderate strength between Facebook ratings and HCAHPS results [13,25,52]. Additionally, some studies have discovered correlations between Facebook ratings and other national patient experience metrics [12,53].

Studies involving other social media platforms revealed a moderate to high correlation between social media ratings and conventional patient satisfaction surveys [3,6], although a couple of studies have shown a negative correlation between social media reviews and patient satisfaction surveys or quality indices [2,54].

It was unknown whether social media reviews were incompatible with other established patient satisfaction measures. The mixed results could be explained by the fact that we examined only public or government hospitals or by the fact that our analysis was a nationwide study, whereas previous studies examined only selected states or hospitals. The difference could also be due to our decision to compare Facebook reviews only to traditional patient satisfaction surveys rather than to Twitter or other social media platforms and multiple clinical quality indicators. Unquestionably, a larger study investigating the connection between social media platforms and hospital quality measures is required. However, there is currently no comparable standard assessment of patient satisfaction or experience in Malaysia’s public or private hospitals. While the MOH favors the SERVQUAL questionnaire, private hospitals may develop their own surveys or use another international standard [55,56]. Thus, Facebook reviews may serve as a new standard of patient satisfaction in both the public and private sectors.

A reviewer’s suggestion in a Facebook review may provide insight into satisfaction with hospital care, which may be useful to other individuals seeking information about hospital quality. Facebook reviews are straightforward and readily accessible, removing barriers to obtaining information about hospital quality and helping hospitals to address quality-of-service concerns and alerting them to possible patient safety issues [15,57]. As a result, a Facebook review may assist both consumers in making healthcare choices and hospitals in ensuring high standards of quality.

Additionally, traditional patient satisfaction surveys are costly, time consuming, have low response rates, necessitate a significant amount of time between hospitalization and public disclosure of reports, frequently fail to identify the source of perceived problems, and may introduce response and selection bias [2,5,11]. The discrepancy between the typical patient survey and other data sources demonstrates the need to use other data sources to ascertain public sentiment about healthcare services [17]. Therefore, the internet in general and social media in particular have been suggested as new tools for evaluating patient satisfaction and monitoring the quality of healthcare services [7,58].

On the other hand, social media evaluations are largely untested and uncontrolled, while conventional patient satisfaction surveys have been validated, assessed, and risk adjusted. Social media users may post information on a hospital or write a review even if they have never been a patient at that hospital. This may also indicate that social media users are leaving reviews or comments on their experiences visiting a friend or family member in the hospital, which is likely related to patient satisfaction with care. More worrisome is that users of social media platforms may post fake reviews [13,24]. To help ensure the authenticity of the data, hospitals may aid customers by posting additional quality metrics on their Facebook sites, using MOH quality indicators, on a rolling six-month basis. This can support the public in making educated choices and encourage the adoption of validated quality measurements.

### 5.2. Hospital Accreditation and Patient Satisfaction

This study provides valuable knowledge regarding patient experiences with healthcare is Southeast Asia, which has not received sufficient attention in previous research. It is a matter of concern that only a few studies have examined patient experience and the impact of accreditation in various Asian contexts, revealing healthcare objectives that vary from those found in the West [59,60].

In general, we found that, after controlling for hospital location and prior Facebook ratings, patient satisfaction in Facebook reviews were not significantly associated with whether a hospital was accredited. Previous research has shown that accreditation has little effect on the quality of treatment received by patients and may not be the most important factor affecting patient desire to recommend hospital services [35,41]. This view is supported by studies in the United States and Germany that found no difference in the ratings or recommendations of accredited and non-accredited hospitals [42,44]. Additional studies in Lebanon [43], Turkey [61], India [62], and Malaysia [63] have all echoed this result. On the other hand, some research has shown a positive relationship between accreditation and patient satisfaction [46,47,48].

There are many possible explanations for the inconsistency in the relationship between patient satisfaction and accreditation. While a focus on patient outcomes is unquestionably beneficial, it is possible that the accreditation process places a greater emphasis on organizational structure, patient safety, and clinical qualities [1,64,65]. The most recent systematic study discovered a link between accreditation and efficiency, effectiveness, timeliness, and safety [32]. Meanwhile, other research has shown a connection between accreditation and clinical outcome improvements such as decreased standardized mortality ratios for chronic illnesses [66,67] and other measures of service quality [68]. However, other studies have shown no correlation between accreditation and clinical outcomes [59,69].

The data provide insight into the relationship between accreditation and its process and results in various areas of the globe. While enhanced clinical procedures may result in improved patient outcomes, it is critical to evaluate hospital activities that can actually increase patient satisfaction [1,59,68]. According to a systematic review, the strongest predictors of patient satisfaction are interpersonal skills and technical care [38]. Additionally, many studies have shown that hospitals with higher clinical quality and/or those that meet accreditation performance criteria, such as reduced readmission rates, have a favorable impact on patients’ overall satisfaction and are therefore highly appreciated by patients, families, and the public at large. Patient satisfaction and Facebook ratings both increased as a result of this appreciation [9,25].

Other factors affecting the connection between accreditation and patient satisfaction include the organization’s features and accessibility, which include size, type, structure, culture, and purpose [25,60,70]. A hierarchical culture has been shown to be associated with reduced readmission rates and reducing readmission rates has a beneficial effect on patient satisfaction. In other words, hierarchical culture is strongly associated with increased patient satisfaction, as has been shown by improving Facebook ratings [9,25]. Additionally, we discovered a strong connection between tertiary hospital type and patient satisfaction, even though some research indicates that only medium-sized hospitals will observe an increase in the quality of their care [68,71].

Patient-related factors were associated with patient satisfaction either weakly or in a mixed fashion. Age, gender, education, socioeconomic position, relationship status, ethnicity, religion, geographic features, frequency of visits, duration of stay, health condition, personality, and expectations are all considered [38]. As proof, we discovered a significant relationship between patient satisfaction and hospital location in an urban region. However, prior research indicates that rural residents were more likely to be pleased than urban residents [38,44]. Additionally, there were little data to substantiate hospital recommendations about hospital accreditation in an urban region [42]. While hospitals situated in urban areas often offer a number of advantages in terms of resources, finances, expertise, and personnel sufficiency, they also come with higher costs and with increased expectations from patients.

Managing patient expectations is inherently challenging. Although theory holds that people are pleased when their expectations match healthcare performance, associations between expectations and satisfaction have varied in published research [38]. Healthcare practitioners, patients, and their families have higher expectations and impressions of patient safety and service quality at accredited hospitals, according to studies [71,72]. Additionally, patients admitted to non-accredited hospitals expressed greater satisfaction with laboratory work, such as professionalism, than patients admitted to accredited hospitals [48].

Moreover, we were unable to identify other patient-related factors, such as age, gender, education, socioeconomic position, relationship status, ethnicity, religion, frequency of visits, duration of stay, health condition, and personality, as key variables in this research. We did not check our reviewers’ Facebook accounts to avoid breaching the Malaysia Personal Data Protection Act or other laws. Age has been shown to have a direct and positive effect on patient satisfaction and service quality rating [43,46]. This finding was echoed by a Malaysian government survey which discovered that, between 2018 and 2020, younger people account for the majority of social media users in Malaysia [21,73]. Other variables, such as gender, can have an effect on patient satisfaction, with male patients expressing higher levels of satisfaction [46]. However a systematic study found inconsistent correlations with the gender factor [38]. A survey found that the majority of internet users in Malaysia are male [21,73], while a study focused on Malaysia discovered no significant difference in gender and patient satisfaction between accredited and non-accredited hospitals [63].

### 5.3. Implications

Our research demonstrates the value of using social media to gather input on facilities and the quality of healthcare services. Social media may offer insights for healthcare organizations that can be used as real-time early-warning signs of a potential decline in healthcare quality or poor patient experiences. It may also be possible to incorporate social media ratings into existing MOH report cards for public hospitals or use them as a supplementary tool for conventional patient satisfaction surveys. Additionally, our study extends the role of hospital administrators and public health organization in enhancing healthcare service quality beyond ongoing monitoring of social media trends for health education or crisis communications. Our findings also encourage all public hospitals in Malaysia to establish and actively engage with the online community through official Facebook pages, given the intangible financial and educational benefits of Facebook pages.

### 5.4. Recommendation

There is a dearth of research on the use of Facebook and other social media platforms in healthcare quality evaluation processes such as accreditation. Malaysia and other developing nations are notable for the slow pace at which healthcare professionals establish and use official Facebook pages. A particularly important area of study would be to examine the variables that promote or inhibit the adoption of official hospital Facebook accounts. This survey should cover all hospital employees and administrators. The information gathered should include hospital workers’ and leaders’ attitudes about and opinions of the creation and use of social media sites. Additionally, we recommend that hospital administrators take Facebook sites and their use more seriously. Because potential patients are likely to form opinions based on social media content, hospitals must approach the service quality of their operations holistically to enhance their social media presence.

Additional research should be conducted to determine how Facebook reviews can be integrated into external measurement systems, including how patient experience scores can be linked to Facebook reviews, how their ambiguity can be addressed, how data changes can be quantified, and how qualitative Facebook data can be interpreted and used. While previous studies have used sentiment analysis, more research should be conducted to determine how to use qualitative data beyond the quantity of positive sentiments. Additional research is needed to obtain a better understanding of the patient satisfaction viewpoint expressed on social media regarding both accredited and non-accredited hospitals. According to the findings of a Lebanese study, tangible hospital characteristics such as physical facilities and equipment have an effect on patient satisfaction [43], a result that has been confirmed by other research [45,61]. Other patient perspectives or quality domains that contribute to patient satisfaction include emergency and inpatient care, triage length, and respect for patients [59,74].

### 5.5. Study Limitations

While our study of Facebook reviews may have been subject to response and selection bias, this is true of any conventional survey. Because the study was conducted in a cross-sectional fashion, we cannot rule out the potential of a causal relationship in our results. Further study on the development of these results would be beneficial. Additionally, only 45 of 87 hospitals had Facebook reviews. The inclusion of unofficial Facebook sites for public hospitals may result in disparate patient satisfaction ratings. Additionally, since the median number of reviews was only 15, many hospitals’ Facebook reviews were insufficient to provide a meaningful indicator of how accreditation affects a hospital’s quality and how it is linked to patient satisfaction on social media. Finally, owing to regulatory and legal constraints, we were unable to examine the effects of accreditation and patient satisfaction on patient-related characteristics. The study of such factors is likely to be beneficial and may provide a richer context for the use of social media in the healthcare sector.

## 6. Conclusions

Despite the fact that more than half of Malaysia’s public hospitals have an official Facebook page, only a handful allow patient feedback in the form of Facebook reviews. As a result, hospital managers are urged to make use of the Facebook review function and leverage its potential as an early-warning system and real-time monitor of hospital quality and patient care. In the present study, we discovered a modest and significant correlation between MOH patient satisfaction survey results and online patient satisfaction as determined by Facebook reviews. Thus, Facebook reviews may be used in conjunction with traditional patient satisfaction surveys. Additionally, we found that accredited hospitals did not achieve a higher level of patient satisfaction on the social media platform than non-accredited hospitals. Although this research found only a modest impact of accreditation on patient satisfaction, accreditation standards are nonetheless internationally acknowledged and should be followed consistently to ensure hospital clinical and quality services. Meanwhile, further research on patient perceptions of patient satisfaction and treatment quality would benefit the healthcare sector. Finally, more reviews are necessary to represent the community of internet users and to obtain a better understanding of the impact of hospital accreditation on online patient satisfaction.

## Figures and Tables

**Table 1:** Hospital and Facebook (FB) characteristics (*n* = 48).

Variable		*n*	(%)	Median	(IQR)
Hospital Characteristics					
Region					
	North	10	(20.8)		
	West	6	(12.5)		
	South	11	(22.9)		
	East Coast	10	(20.8)		
	Borneo	11	(22.9)		
Type of Hospital					
	Primary	26	(54.2)		
	Secondary	4	(8.3)		
	Tertiary	18	(37.5)		
Location					
	Rural	22	(45.8)		
	Urban	26	(54.2)		
Number of Beds				730	(563)
Average Patient Satisfaction in MOH Survey				96.93	(3.00)
Hospital with Accreditation Status					
	No	23	(47.92)		
	Yes	25	(52.08)		
FB Characteristics					
Previous FB Star Ratings				5.00	(1.65)
Number of Reviews				15.5	(27.5)
Adequate Hospital Information on FB Page					
	No	11	(22.9)		
	Yes	37	(77.1)		
Hospital Administration Replied to FB Reviews					
	No	18	(37.5)		
	Yes	30	(62.5)		
Average Proportion of Patient Recommendation from FB Review				80.7	(48.43)

**Table 2:** Facebook reviews and their characteristics (*n* = 2019).

Variables		*n*	(%)
Hospital Characteristics			
Region			
	East Coast	219	10.8
	North	441	21.8
	West	992	49.1
	South	202	10.0
	East Malaysia	165	8.2
Location			
	Rural	261	12.9
	Urban	1758	87.1
Type of Hospital			
	Primary	136	6.7
	Secondary	96	4.8
	Tertiary	1787	88.5
Accreditation Status			
	No	783	38.8
	Yes	1236	61.2
FB Page Characteristics			
Hospital Administration Response			
	No	1836	90.9
	Yes	183	9.1
Patient Recommendation	No	517	25.6
	Yes	1502	74.4

**Table 3:** Factors associated with patient satisfaction (*n* = 2019).

Variables		*B*	Crude OR	95% (Lower)	CI (Upper)	*p*-Value *
Hospital Characteristics						
Region	East Coast		Ref			
	North	0.51	1.66	1.12	2.47	0.013
	West	0.03	1.03	0.73	1.44	0.877
	South	−0.61	0.54	0.34	0.83	0.004
	East Malaysia	−0.71	0.49	0.32	0.76	0.001
Location	Rural		Ref			
	Urban	0.61	1.85	1.40	2.43	<0.001
Hospital Type	Primary		Ref			
	Secondary	0.10	1.11	0.64	1.93	0.725
	Tertiary	0.48	1.62	1.12	2.35	0.014
Numbers of Bed		1.19	1.00	1.00	1.00	0.273
FB Page Features						
Previous FB Rating		0.13	1.14	1.06	1.23	<0.001
Adequate Hospital Information on FB Page						
	No		Ref			
	Yes	0.25	1.28	0.85	1.92	0.232
Hospital Administration Reply						
	No		Ref			
	Yes	−0.27	0.76	0.55	1.06	0.114
Hospital Accreditation Status						
	No		Ref			
	Yes	0.03	1.03	0.84	1.26	0.791

* Simple Logistic Regression.

**Table 4:** Factors associated with patient satisfaction using multivariate analysis (*n* = 2019).

Variables		Adjusted OR	95 % CI		*p*-Value *
			(Lower)	(Upper)	
Accreditation	No	Ref			
	Yes	0.95	0.77	1.17	0.633
Hospital Location	Rural	Ref			
	Urban	1.71	1.29	2.27	<0.001
Previous FB Rating		1.11	1.03	1.20	0.014

* Multiple Logistic Regression; Constant = 0.203; Forward LR, backward LR, and manual selection were applied for the confirmatory analysis. No significant interaction or multicollinearity. Hosmer and Lemeshow Test = 0.10. Classification Table = 74.4%. Area under the ROC Curve = 58% (*p* < 0.001).

## Data Availability

The Facebook data presented in this study are available on request from the corresponding author. The data are not publicly available due to privacy. However, restrictions apply to the availability of hospital data. Data was obtained from Ministry of Health and are available from the authors with the permission of Ministry of Health.
